# An fMRI Dataset on Social Reward Processing and Decision Making in Younger and Older Adults

**DOI:** 10.1038/s41597-024-02931-y

**Published:** 2024-02-01

**Authors:** David V. Smith, Rita M. Ludwig, Jeffrey B. Dennison, Crystal Reeck, Dominic S. Fareri

**Affiliations:** 1 Temple University, Philadelphia, PA USA; 2 University of Pennsylvania, Philadelphia, PA USA; 3 Adelphi University, Garden City, NY USA

**Keywords:** Cognitive ageing, Motivation, Human behaviour, Cooperation

## Abstract

Behavioural and neuroimaging research has shown that older adults are less sensitive to financial losses compared to younger adults. Yet relatively less is known about age-related differences in social decisions and social reward processing. As part of a pilot study, we collected behavioural and functional magnetic resonance imaging (fMRI) data from 50 participants (Younger: N = 26, ages 18–34 years; Older: N = 24, ages 63–80 years) who completed three tasks in the scanner: an economic trust game as the investor with three partners (computer, stranger, friend) as the investee; a card-guessing task with monetary gains and losses shared with three partners (computer, stranger, friend); and an ultimatum game as responder to three anonymous proposers (computer, age-similar adults, age-dissimilar adults). We also collected B_0_ field maps and high-resolution structural images (T_1_-weighted and T_2_-weighted images). These data could be reused to answer questions about moment-to-moment variability in fMRI signal, representational similarity between tasks, and brain structure.

## Background & Summary

Close relationships have powerful influences over our lives, supporting physical and emotional well-being and fulfilling social needs to connect. Interactions with close others drive many decisions by altering the value placed on the outcomes of choices (e.g., choosing healthy vs. unhealthy eating habits). Evidence from our group indicates that the social context created by the presence of a friend enhances responses within the brain’s reward circuit and influences collaborative decisions in young adults^1,2^. Yet, it remains unclear whether the effects of social context on reward-based decision making change between younger and older adulthood. This is a key outstanding question in the literature, considering that older adults are frequently the targets of financial exploitation, and financial exploitation yields annual losses of nearly $3 billion with the vast majority of cases being perpetrated by strangers (51%) or friends and family (34%)^3^. Thus, understanding how older adults integrate information from the social domain to inform economic decision making could have important implications for both understanding the nature of social influence on decision making across the lifespan and for crafting social interventions to improve social decisions in older adulthood.

To address these gaps in the literature, we recruited 50 participants (ages 18 to 80) who engaged in three tasks involving social and economic decision making while having brain responses measured with functional magnetic resonance imaging (fMRI). Since our primary focus was how participants trust others, each participant first completed a modified economic trust game where they acted as the investor with either a friend, a stranger, or a computer acting as the investee^2,4^. Next, participants completed a guessing game^5^ where the monetary outcomes were shared with either a friend, a stranger, or a computer^1^. Finally, participants completed a modified ultimatum game^6^ as the recipient who was playing with anonymous proposers who were either age-similar, age-dissimilar, or a computer. These tasks were chosen because they allowed the examination of how social context impacts economic decision making. In addition to these scanner tasks, participants also completed a short battery of personality and cognitive assessments. We also collected high-resolution structural images emphasizing T1-weighted (T1w) and T2-weighted (T2w) contrast.

Although we have previously published results from this dataset relating to the trust game^4^, we have not published results relating to the other two tasks and we believe there are several opportunities for reuse and extension. First, the structure of our study—i.e., multiple tasks tapping into social and economic decision making—would be amenable to secondary analyses that seek to assess associations across tasks. For example, future work using these data could assess similarity across tasks using representational similarity analyses or multivariate pattern analyses^7–10^. In a similar vein, future work that uses these data could examine how responses in one task (e.g., ventral striatal response to reward relative to punishment) are associated with responses in another task (e.g., dorsolateral prefrontal cortex response to rejecting unfair offers) and further test how such cross-task associations differ across age groups. Second, we collected both T1w and T2w images, thus permitting researchers to construct myelin maps^11–13^. These structural metrics could be combined with functional responses and provide novel directions to examine age-related differences in brain function. Finally, given that our data is in the brain imaging data structure (BIDS) format^14^ and openly available on OpenNeuro.org^15–17^, we believe it can be easily combined with other datasets. Such efforts would provide enhanced power to detect age-related differences in brain structure and function and also provide additional power for broader methodological issues, including the functional significance of moment-to-moment variability in the BOLD signal^18,19^. Taken together, these examples underscore the value of our data and its potential utility.

## Methods

### Participants

**Fig. 1:**
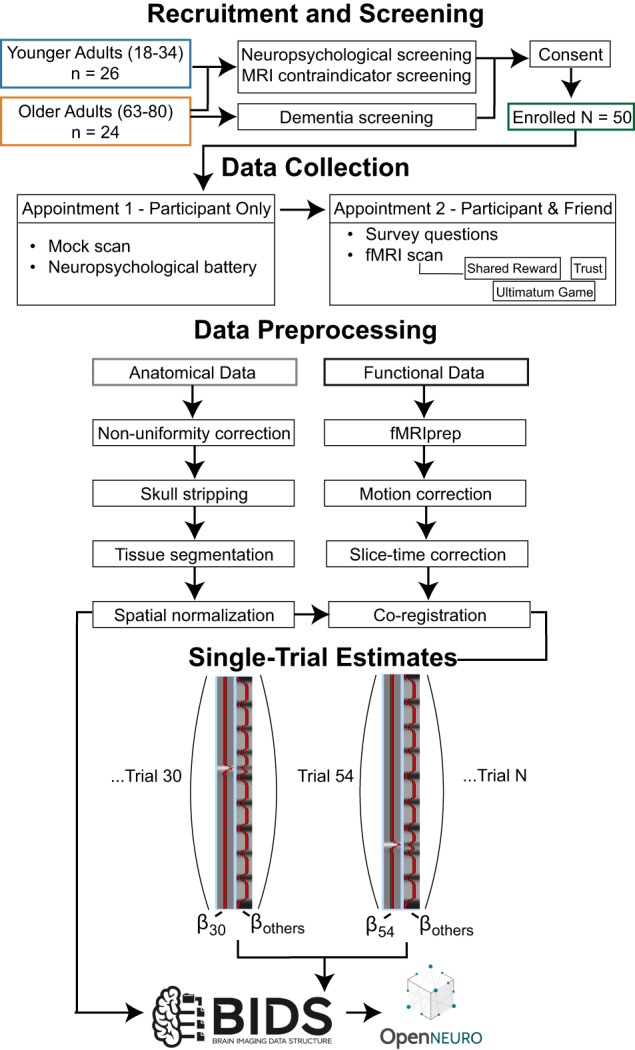
A schematic of the experiment workflow and data preprocessing. Depicts how the data were handled from participant recruitment to deposition on OpenNeuro in BIDS format. Single-trial estimates of activation were derived from the functional data using a Least Squares – Separate (LSS) approach.

As described in our previous work^4^, we recruited 50 participants (26 young adults, ages 18–34 years; 24 older adults, ages 63–80 years) to participate in this pilot study of age-related differences in social and economic decision making (see Fig. 1 for full workflow). We identified young adult participants through the Temple University Psychology Department participant pool and local community. Older adult participants were recruited using a range of efforts, including reaching out to local community and senior centres, social media, newspaper advertisements, and local flyers. Participants recruited through the departmental participant pool were compensated for their time with course credit, while participants from the community were compensated with Amazon and/or Visa gift cards ($25 per hour of participation for MRI participants, $15 per hour for behavioural participants). To ensure that choices were incentive-compatible and that participants were motivated to engage in the tasks, we also provided additional bonus payments based on randomly chosen outcomes paid out at the end of the experimental session.

All participants were screened before data collection to rule out current major psychiatric or neurologic illness, as well as MRI contraindications. In addition, participants who could not attend the scanning session with a friend were also excluded. Older adults were screened to rule out dementia using the Telephone Interview for Cognitive Status, with a score under 30 meeting exclusion criteria^20^.

All participants provided written informed consent to engage in the study and share their de-identified data publicly. The study was approved by the Institutional Review Board at Temple University (Philadelphia, Pennsylvania, USA) under Protocol Number 24452.

### Procedures

Following recruitment and eligibility screening, each participant completed two in-person appointments. During the first appointment, participants underwent a mock MRI scan to help rule out claustrophobia and acclimate to the scanner. The mock scan allowed us to train participants to remain still in the scanner environment while performing a task. During the first appointment, older participants completed a brief neuropsychological test battery including the Functional Activities Questionnaire (FAQ) for exclusion purposes (FAQ > 9).

Participants completed a second appointment with a self-selected friend of the same identified sex and age group (within 5–10 years), who was not a family member, spouse or romantic partner. During the second appointment, participants completed a 90-minute MRI session with the experimental tasks (see below). Due to experimenter error, additional neuropsychological data and demographic data beyond age and gender are not available for the present dataset.

### Social and economic decision-making tasks

**Fig. 2:**
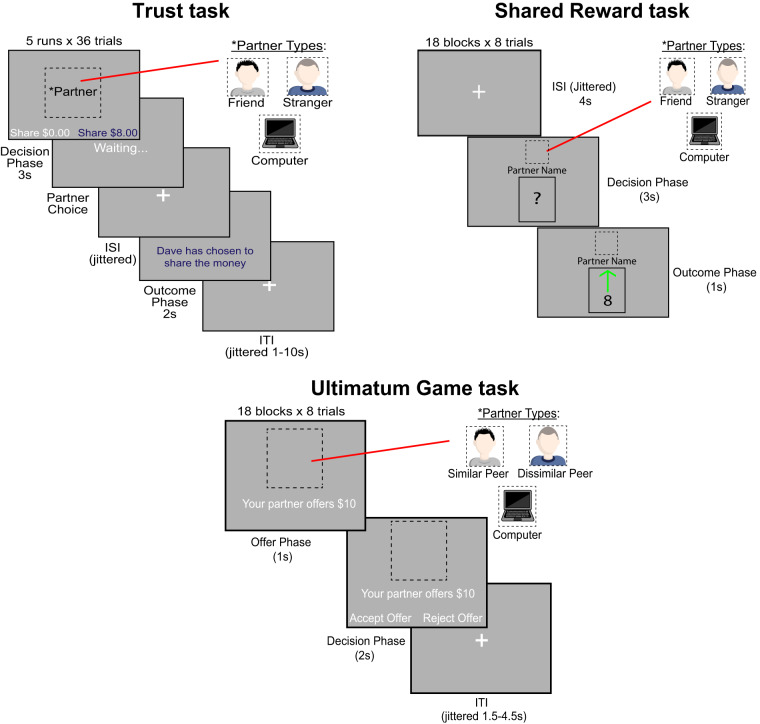
A schematic of the Shared Reward, Trust, and Ultimatum Game tasks. Participants played three economic games with varying social conditions while undergoing fMRI.

Participants completed three tasks while undergoing fMRI–an iterated trust game^2,4^, a shared reward processing task^4^, and an Ultimatum Game task (Fig. 2). The order of tasks was fixed across participants (Trust Game, Shared Reward Paradigm, Ultimatum Game). To maximize engagement and ensure incentive compatible decisions, participants were instructed that payment from each task would be based on a randomly chosen trial.

Each task was played with partners. In the Trust Game and Shared Reward tasks, the stranger/confederate was a sex-matched and age-group matched (i.e., within 10 years of the participant) individual who was recruited for participation from the community in a manner similar to that used to recruit the MRI participants and their friends. The recruited strangers attended the experimental sessions and MRI participants and their friends were introduced to them as additional participants in the study. After introduction, the strangers were placed in a room separate from the MRI control room. The MRI participant was under the impression that they would be interacting with both the friend and the stranger in real time in the trust game, and that their choices in the Shared Reward task would impact both their friend’s and the stranger’s bonus compensation. In the Ultimatum game, participants were told that their counterparts were real individuals who had played the game before and indicated the offers that they would like to make.

#### Trust game task

Our previous and related work on this dataset utilized the Trust Game^4^. Thus, the task description from that work has been adapted for this manuscript. Participants undergoing fMRI first completed an iterated trust game task in the role of ‘investor’, modified from prior work^2^ with three different ‘investees’–a close friend, a stranger (i.e., confederate) or a computer (i.e., non-social control condition). On each trial of the task, investors played with one of the three investees, and started out with $8. Participants were told that they would have the opportunity to decide to share some portion of that $8 (i.e., $0, $2, $4, or $8) with the investee on a given trial, and that if they chose to share a non-zero amount, then the money shared would triple for the investee, who could then choose to send back half or nothing to the investor. Investors had up to 3 seconds to indicate how much they wanted to share on a given trial using an MR-compatible fibre optic response box. Upon indicating their response, they were presented with a screen that said ‘Waiting’ (for 1.5 s) and were instructed that their responses were being transmitted in real-time to a given investee, who was in turn making their choice to share back or not. After a variable ISI following the ‘Waiting’ screen (mean 1.42 s), investors were then provided feedback for 2 s as to what their partner’s choice was. Unbeknownst to participants, all investees reciprocated trust at a 50% rate, consistent with prior work (e.g.^1,2^). All trials were separated by a jittered ITI (ranging from 2–8 s).

Depending on how much time remained in the scanning session, we administered up to five runs of the trust game task. Each run consisted of 36 trials in total (12 per partner), for a maximum of 180 trials, 60 per partner. We note that all 50 participants completed at least two runs of the task, while 42 participants completed three runs, 40 participants completed 4 runs, and 38 participants completed all five runs.

#### Shared reward task

Participants also performed a second task–a card guessing game for shared monetary outcomes (cf.^1^)–with three partners: the same close friend that accompanied them to the experimental session from the trust game, the same confederate, and the computer. In this task, participants were told that they were going to play a game in which their goal on each trial was simply to guess whether the numerical value of a card would be higher or lower than the number 5. On each trial of the task, a card was displayed with a question mark inside for up to 3 seconds. At the top of the screen, an image of the participant’s partner for that trial was also displayed, so they were aware of with whom they would be sharing monetary wins and losses. Participants would press a designated button on the MR-compatible response box to enter a guess of lower than 5, and another button to enter a guess of higher than 5. Upon entering their response, the question mark would highlight orange for 0.5 seconds before a short ISI (0.75 s) was presented. After the ISI, participants were presented with the numerical value of the card on that trial, as well as a green check mark indicating a correct response, or a red ‘X’ indicating an incorrect response. Correct guesses were associated with a monetary gain of $10, shared with a partner on a given trial; incorrect guesses were associated with a monetary loss of $5, shared with a partner on a given trial.

Trials were presented in a block design. All blocks were separated by an 8–12 second between block interval. Within each block, participants played a series of eight trials with one partner (I.e., friend, stranger or computer). Further, each block consisted of either mostly reward trials (75%) or mostly loss (75%) trials, mirroring the structure from the Human Connectome Project^21^. Interspersed within each block was one trial of the opposite outcome valence and one neutral outcome (card value equivalent to 5, no monetary gain or loss associated with these trials). There were a total of 2 runs of 9 blocks each (18 blocks total, 3 Reward and 3 Loss for each partner); each block consisted of 8 trials in total (total number of trials = 144).

#### Ultimatum game task

Last, we administered an iterated Ultimatum Game task in order to assess perceptions of and responses to (un)fair offers from others. Here, all participants played in the role of responders in the Ultimatum Game and played with three different proposers—a computer (non-social control), a same-gender, younger adult peer and a same-gender older adult peer. We also matched on self-reported race (i.e., white, non-white). For young adult participants, the young adult peer was considered a ‘similar’ peer, while the older adult peer was a ‘dissimilar’ peer. The converse was true for older adult participants. All ‘human’ peers were depicted using younger and older-looking emojis. The computer partner was depicted using the same computer image used in prior tasks in this dataset.

Participants played with one partner in each block of a block design version (8 trials per block) of the Ultimatum Game. On each trial, a given proposer started out with $20, which they could decide to split with the participant in any way they wanted. Participants were presented with the proposer’s offer for 1 second and had an additional 2 seconds to indicate whether they wanted to accept or reject that offer. Participants were told that if they rejected the offer, neither they nor the proposer on a trial would receive anything for that trial. Unbeknownst to participants, proposer behaviour was predetermined: blocks consisted of either majority fair (i.e., 6 out of 8 offers were for between 35%–50% of the $20) or majority unfair offers (i.e., 6 out of 8 offers were for between 5% and 20% of the $20). In both block types, the remaining two trials consisted of one neutral offer (25% or 30% of the $20) and one offer from the other condition, thus mirroring the block structure used in the Human Connectome Project^21^. In total, 18 blocks of trials were presented (144 trials in total), 3 fair blocks and 3 unfair blocks for each proposer type.

### Neuroimaging data collection

The following description of neuroimaging scanning parameters is adapted from our previous work on this dataset (Fareri *et al*., 2022). We collected neuroimaging data at the Temple University Brain Research and Imaging Centre (TUBRIC) using a 3.0 Tesla Siemens Prisma scanner and a 20-channel phased-array head coil. Functional images sensitive to blood-oxygenation-level-dependent (BOLD) contrast were collected using a single-shot T2*-weighted echo-planar imaging sequence with slices roughly parallel to the axial plane collected in descending order [repetition time (TR): 2.02 s; echo time (TE): 23 ms; matrix 74 × 74; voxel size: 2.97 × 2.97 × 2.80 mm; 36 slices (15% gap); flip angle: 76°]. We also collected other images to facilitate co-registration and normalization of functional data and allow for other analyses focused on age-related structural differences. These additional images included the following types of scans: a high-resolution T1-weighted structural scan (TR: 2.4 s; TE: 2.2 ms; matrix 192 × 192; voxel size: 1.0 mm^3^; 192 slices; flip angle: 8°); a B0 field map (TR: 645 ms; TE1: 4.92 ms; TE2: 7.38 ms; matrix 74 × 74; voxel size: 2.97 × 2.97 × 2.80 mm; 36 slices, with 15% gap; flip angle: 60°); and a T2-weighted structural images (TR: 3.2 s; TE: 567 ms; matrix 192 × 192; voxel size: 1.0 mm^3^; 192 slices; flip angle: 120°).

### Neuroimaging data processing

DICOMs were first converted to NIFTI form using dcm2niix^22^ and organized into the Brain Imaging Data Structure^14^ using HeuDiConv (https://github.com/nipy/heudiconv).

Results included in this manuscript come from preprocessing performed using fMRIPrep 21.0.2^23^, which is based on Nipype 1.6.1^24^.

#### Preprocessing of B0 inhomogeneity mappings

A B0 nonuniformity map (or fieldmap) was estimated from the phase-drift map(s) measure with two consecutive GRE (gradient-recalled echo) acquisitions. The corresponding phase-map(s) were phase-unwrapped with prelude (FSL 6.0.5.1:57b01774).

#### Anatomical data preprocessing

A total of 1 T1-weighted (T1w) images were found within the input BIDS dataset. The T1-weighted (T1w) image was corrected for intensity non-uniformity (INU) with N4BiasFieldCorrection^25^, distributed with ANTs 2.3.3^26^ and used as T1w-reference throughout the workflow. The T1w-reference was then skull-stripped with a Nipype implementation of the antsBrainExtraction.sh workflow (from ANTs), using OASIS30ANTs as target template. Brain tissue segmentation of cerebrospinal fluid (CSF), white-matter (WM) and gray-matter (GM) was performed on the brain-extracted T1w using fast (FSL 6.0.5.1^27^). Brain surfaces were reconstructed using recon-all (FreeSurfer 6.0.1^28^), and the brain mask estimated previously was refined with a custom variation of the method to reconcile ANTs-derived and FreeSurfer-derived segmentations of the cortical gray-matter of Mindboggle^29^. Volume-based spatial normalization to two standard spaces (MNI152NLin2009cAsym, MNI152NLin6Asym) was performed through nonlinear registration with antsRegistration (ANTs 2.3.3), using brain-extracted versions of both T1w reference and the T1w template. The following templates were selected for spatial normalization: ICBM 152 Nonlinear Asymmetrical template version 2009c^30^; [TemplateFlow ID: MNI152NLin2009cAsym], FSL’s MNI ICBM 152 non-linear 6th Generation Asymmetric Average Brain Stereotaxic Registration Model^31^; [TemplateFlow ID: MNI152NLin6Asym].

#### Functional data preprocessing

For each of the BOLD runs found per subject (across all tasks and sessions), the following preprocessing was performed. First, a reference volume and its skull-stripped version were generated using a custom methodology of fMRIPrep. Head-motion parameters with respect to the BOLD reference (transformation matrices, and six corresponding rotation and translation parameters) are estimated before any spatiotemporal filtering using mcflirt (FSL 6.0.5.1^32^. The estimated fieldmap was then aligned with rigid-registration to the target EPI (echo-planar imaging) reference run. The field coefficients were mapped on to the reference EPI using the transform. BOLD runs were slice-time corrected to 0.972 s (0.5 of slice acquisition range 0s-1.95 s) or 0.971 s (0.5 of slice acquisition range 0s-1.94 s) using 3dTshift from AFNI^33^. The BOLD reference was then co-registered to the T1w reference using bbregister (FreeSurfer) which implements boundary-based registration^34^. Co-registration was configured with six degrees of freedom.

Several confounding time-series were calculated based on the preprocessed BOLD: framewise displacement (FD), DVARS and three region-wise global signals. FD was computed using two formulations following Power (absolute sum of relative motions^35^) and Jenkinson (relative root mean square displacement between affines^32^,). FD and DVARS are calculated for each functional run, both using their implementations in Nipype (following the definitions by^35^). The three global signals are extracted within the CSF, the WM, and the whole-brain masks. Additionally, a set of physiological regressors were extracted to allow for component-based noise correction (CompCor^36^). Principal components are estimated after high-pass filtering the preprocessed BOLD time-series (using a discrete cosine filter with 128 s cut-off) for the two CompCor variants: temporal (tCompCor) and anatomical (aCompCor). tCompCor components are then calculated from the top 2% variable voxels within the brain mask. For aCompCor, three probabilistic masks (CSF, WM and combined CSF + WM) are generated in anatomical space. The implementation differs from that of Behzadi *et al*. in that instead of eroding the masks by 2 pixels on BOLD space, the aCompCor masks are subtracted a mask of pixels that likely contain a volume fraction of GM. This mask is obtained by dilating a GM mask extracted from the FreeSurfer’s aseg segmentation, and it ensures components are not extracted from voxels containing a minimal fraction of GM. Finally, these masks are resampled into BOLD space and binarized by thresholding at 0.99 (as in the original implementation). Components are also calculated separately within the WM and CSF masks. For each CompCor decomposition, the k components with the largest singular values are retained, such that the retained components’ time series are sufficient to explain 50 percent of variance across the nuisance mask (CSF, WM, combined, or temporal). The remaining components are dropped from consideration. The head-motion estimates calculated in the correction step were also placed within the corresponding confounds file. The confound time series derived from head motion estimates and global signals were expanded with the inclusion of temporal derivatives and quadratic terms for each^37^. Frames that exceeded a threshold of 0.5 mm FD or 1.5 standardised DVARS were annotated as motion outliers.

The BOLD time-series were resampled into standard space, generating a preprocessed BOLD run in MNI152NLin2009cAsym space. First, a reference volume and its skull-stripped version were generated using a custom methodology of fMRIPrep. The BOLD time-series were resampled onto the following surfaces (FreeSurfer reconstruction nomenclature): fsaverage. Grayordinates files^38^ containing 91k samples were also generated using the highest-resolution fsaverage as intermediate standardized surface space. All resamplings can be performed with a single interpolation step by composing all the pertinent transformations (i.e. head-motion transform matrices, susceptibility distortion correction when available, and co-registrations to anatomical and output spaces). Gridded (volumetric) resamplings were performed using antsApplyTransforms (ANTs), configured with Lanczos interpolation to minimize the smoothing effects of other kernels^39^. Non-gridded (surface) resamplings were performed using mri_vol2surf (FreeSurfer).

Many internal operations of fMRIPrep use Nilearn 0.8.1^40^, mostly within the functional processing workflow. For more details of the pipeline, see the section corresponding to workflows in fMRIPrep’s documentation.

### Quality assurance

Behavioural data were assessed for quality by examining missed trials. A large number of missed trials would indicate task non-compliance (e.g., sleeping) and/or error in data collection.

For the neuroimaging data, a set of image quality metrics were generated using MRIQC version 22.0.6^41^. The provided image quality metrics can be used to exclude outlier runs and subjects or as covariates for higher levels of analysis. This can be especially useful when there are differences between groups of interest in motion and other aspects of image quality that can induce spurious results^42^. Nevertheless, we note that the relationship between head motion and changes in BOLD are complicated and nuanced^43–47^.

We note that no data were excluded from the published dataset. Thus, users must make their own decisions regarding exclusions.

### Generation of single-trial estimates

For additional convenience of researchers wanting to use methods taking advantage of multivoxel pattern analysis and similar methods, we have provided single trial estimates of the BOLD response. Each trial estimate image was estimated by a unique trial model, based on the approach called Least-squares Single (LSS). Using the LSS approach has been shown to reduce collinearity between estimates, especially for designs with short intertrial intervals^48–50^.

Neuroimaging analyses were conducted using FEAT (FMRI Expert Analysis Tool) version 6.00^51^. The bold response for each image was estimated using a general linear model with local autocorrelation correction^52^ consisting of two regressors one reflecting the response to the trial being estimated and another for the BOLD responses to all trials *except* the trial being estimated. This approach returns two parameter estimates, one for the trial of interest and a nuisance parameter estimate representing the activation for all other trials. We defined the duration of each regressor as the period of time from the presentation of the outcome (reciprocate or defect) for a duration of 1 s. Only data within the same run were included in any model.

Each model contained a set of confounds from the output of fmriprep. These confounds included three translation and rotation motion parameters, non-steady state volumes, a set of cosine basis functions for temporal filtering, as well as the first six anatomical CompCor regressors described above, and measure of framewise displacement. No smoothing was applied prior to analyses and the entire four-dimensional dataset was grand-mean intensity normalized using a single multiplicative factor. Non-brain voxels in the functional images were removed using the Brain Extraction Tool^53^.

## Data Records

All data records are organized according to the BIDS (Brain Imaging Data Structure) standard (version 1.4.1). Structural and functional neuroimaging data are publicly available as OpenNeuro Dataset ds003745^54^. Each participant’s data are organized within participant-specific directories with the naming scheme <sub-XXX>, and each directory contains three subdirectories. Structural data are stored within an ‘anat’ directory; functional data are stored within a ‘func’ directory, and B0 fieldmaps are stored within an ‘fmap’ directory. Task-relevant behavioural data are also stored in each participant’s ‘func’ directory as *events.tsv files.

In addition to the raw data, the dataset also includes preprocessed data using FMRIprep, quality assurance metrics using MRIQC, and single-trial estimates generated by FSL. These derivatives are stored under the ‘derivatives’ subdirectory on OpenNeuro.

The entire dataset is openly available via OpenNeuro (10.18112/openneuro.ds003745.v2.1.1).

## Technical Validation

We carried out several procedures to ensure the technical validation of our behavioural data, as well as our structural and functional neuroimaging data. For each type of data, we present results across all participants, and we also present age-related differences. Although age-related differences may be a focus of some secondary analyses, other studies may wish to control for such differences. The results of these validations are presented below. We note that all validations are presented with the full dataset. Users can make their own determinations about exclusions.

### Behavioural data

Ensuring that each participant has enough trials in each task is paramount for many secondary analyses. We therefore examined the response time data and missed trials for each task. In each task, we observed a small percentage of missed trials (Shared Reward: Older: 12.41%, Younger: 2.78%; Trust Game Older: 5.84% Younger: 2.12%; Ultimatum Game Older: 1.23%, Younger: 0.51%).

We also examined age-related differences in behaviour. We found that older adults (*M* = 6.24%) missed a significantly larger proportion of trials than younger adults (*M* = 1.96%; *t*(48) = 2.63, *p = *0.014). We also found age-related differences in response time. Specifically, older adults were generally slower than younger adults across all tasks (*t*(48) = 2.36, *p = *0.023). We also found that variability in response times differed across age groups such that older adults had more variability than younger adults in all tasks (*t*(48) = 2.32, *p* = 0.025).

### Structural neuroimaging data

**Fig. 3:**
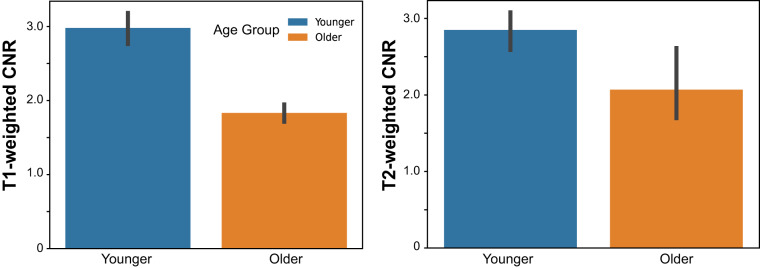
Structural data quality. Temporal contrast-to-noise ratio (CNR) by age group. Positive values indicate greater activation signal than noise. The left panel displays the T1-weighted CNR; the right, the T2-weighted. Error bars reflect 95% confidence intervals.

We validated and assessed the quality of the T1w and T2w structural images using MRIQC. All participants had high contrast-to-noise ratio in both images (see Fig. 3), indicating clear separation between grey matter and white matter tissues. Younger adults had significantly greater contrast-to-noise ratio for the T1w images (*t*(48) = 8.94, *p* < 0.001) and the T2w images (*t*(48) = 2.88, *p = *0.007).

### Functional neuroimaging data

**Fig. 4:**
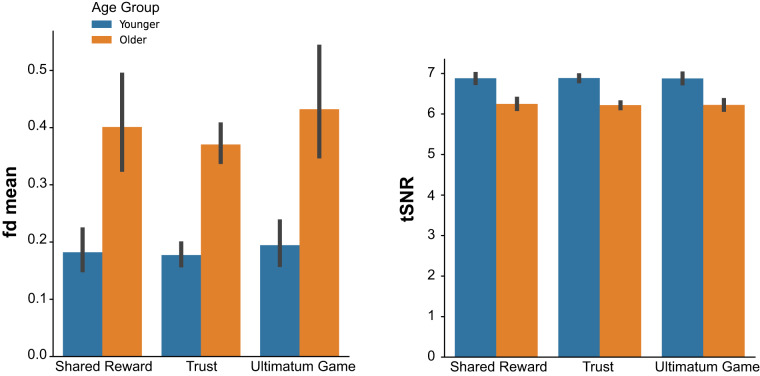
Functional data quality by task and age group. The left panel graphs the mean frame displacement of each run (fd mean), which overall was larger for older participants during each task. The right panel graphs the temporal signal-to-noise ratio (tSNR), which was slightly lower in older participants across all tasks. Error bars reflect 95% confidence intervals.

We first examined the degree of head motion in each task. High amounts of head motion can warrant data exclusions as head motion can compromise quality and validity. We specifically focused on framewise displacement, which summarizes volume-to-volume changes in head motion. Across all tasks, average framewise displacement was low (Fig. 4). However, we observed age-related differences in framewise displacement in each task (Ultimatum Game *t*(48) = 4.31, *p < *0.001; Shared Reward *t*(48) = 4.57, *p < *0.001; Trust Game *t*(48) = 9.71, *p* < 0.001). This observation may warrant the inclusion of framewise displacement covariates in all analyses examining age differences, as done in our prior work^4,55^. Nevertheless, we note that the links between head motion and individual differences in brain responses are complicated^43,44,46^, and researchers must be careful about how they approach this problem in their own work^47,56^.

**Fig. 5:**
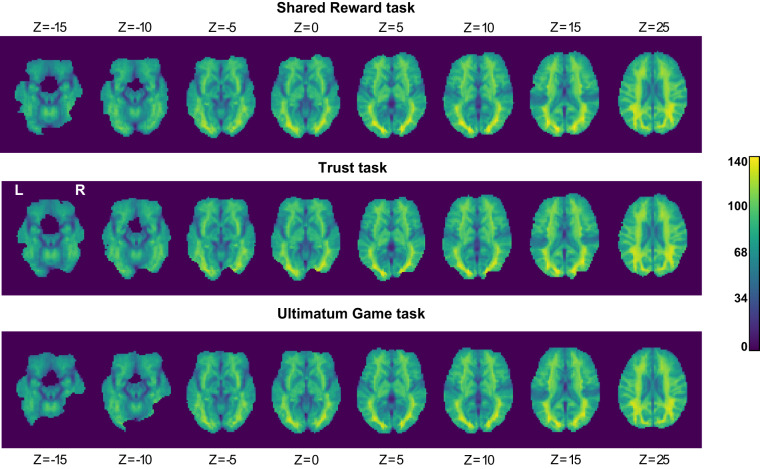
Temporal signal-to-noise ratio (tSNR) by task. High tSNR is indicative of better quality data; here, we find that the tSNR during each task is high and relatively uniform across the whole brain.

Next, we examined the temporal signal-to-noise ratio (tSNR) in each task using MRIQC. Low tSNR is indicative of low-quality and potentially-problematic functional data. We found that the tSNR was high and relatively uniform across the whole brain (Fig. 5). This is important because studies using phased-array headcoils often have drop-offs in tSNR in deep-brain structures like the striatum^21^.

**Fig. 6:**
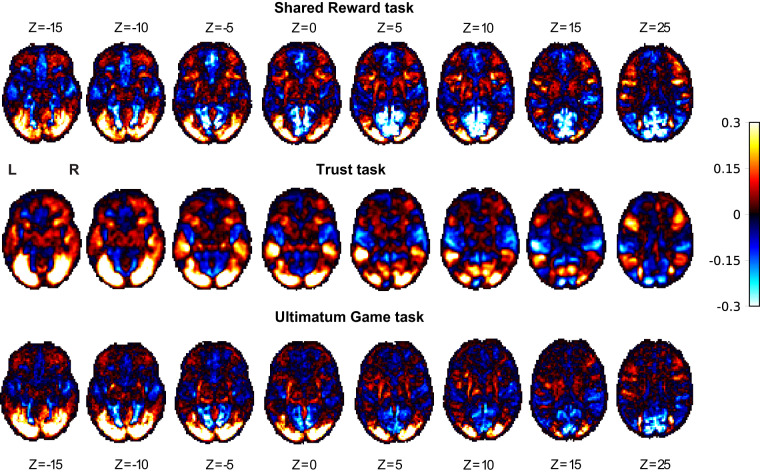
Mean single-trial estimates by task. Single-trial estimates were generated using a Least Squares – Separate (LSS) approach.

Finally, we assessed average task-related activation in our single-trial estimates in each task. If there was an error in our data collection procedures, we would not expect to see task-related responses in visual cortex and motor cortex. Consistent with this prediction, we found that the average response across single trials was associated with increased activation in the visual cortex and motor cortex. We also found that the posterior cingulate cortex was consistently deactivated across tasks, consistent with previous work on the default-mode network^57–59^. We observed a similar pattern for all three tasks (Fig. 6).

## Usage Notes

Data are organized using BIDS naming conventions^14^. We used the following key/pair values “sub-<value>”, “task-<value>”, “run-<value>”. Sub refers to a person participating in the study values are numerical ranging from 104–157, however, these values are not sequential. Task refers to one of the three tasks described in the text and takes one of the following values “trust”,”sharedreward”, or “ultimatum”. Run refers to separate acquisitions of functional data using the same parameters, subject, and task.

The derivatives subfolder has four sub-directories; “fmriprep”, “mriqc”, “single_trials”, “tsnr”. The fmriprep directory contains all of the output from preprocessing using fmriprep as described in the text. The mriqc directory contains the raw image quality metrics and output from mriqc as described in the text. The single_trials directory contains an organized set of single trial estimates for each subject. Each subject’s data is contained in a directory named with the appropriate key-pair. Within each directory is one 4D nifti image which is related to a single run of a single task for each participant with the following naming convention sub-<value>_task-<value>_run-<value>_singletrial-Act.nii.gz. Each volume represents a single trial in order from earliest to latest; however, missed trials are not contained in the single trial image. This means that if a participant missed their response on the first trial, the first volume would correspond to trial two etc. The tsnr directory contains one volumetric image for each participant, task, and run using the naming scheme sub-<value>_task-<value>_run-<value>_tsnr.nii.gz. This image represents the mean signal divided by the standard deviation of the signal throughout the corresponding functional image.

To ensure anonymization, all structural data have been defaced using PyDeface (https://github.com/poldracklab/pydeface). In addition, data collection dates have been shifted.

## Data Availability

All code is available on GitHub without restrictions (https://github.com/DVS-Lab/srndna-datapaper). A snapshot of the repository has been placed on Zenodo for permanent archiving (https://zenodo.org/doi/10.5281/zenodo.10456520). This repository includes a README.md file that describes how the dataset was generated. This repository also includes stimulus presentation scripts and the sourcedata generated by those scripts.
